# Transformed through the CARTA experience: changes reported by CARTA fellows about their PhD journey

**DOI:** 10.1080/16549716.2023.2272392

**Published:** 2023-11-09

**Authors:** Anne Ruhweza Katahoire, Jill Allison, Marta Vicente-Crespo, Sharon Fonn

**Affiliations:** a Child Health and Development Center, Makerere University, Kampala, Uganda; b Faculty of Medicine, Memorial University of Newfoundland, St. John's, Canada; c African Population and Health Research Center, Nairobi, Kenya; d School of Public Health, University of the Witwatersrand, Johannesburg, South Africa; e School of Public Health and Community Medicine, University of Gothenburg, Gothenburg, Sweden

**Keywords:** Early career researcher, Africa, career path, change agents, research leadership

## Abstract

Transformative learning occurs when a person, group, or larger social unit encounters ideas that are at odds with their prevailing perspective. This discrepant perspective can lead to an examination of previously held beliefs, values, and assumptions. The Consortium for Advanced Research Training in Africa (CARTA) has since 2011 been training and supporting faculty from different African universities, to become more reflective and productive researchers, research leaders, educators, and change agents who will drive institutional changes in their institutions. As part of a mid-term evaluation of CARTA, an open-ended question was posed to the CARTA fellows asking them to describe any changes they had experienced in their professional lives as a result of the CARTA Programme. The 135 responses were inductively coded and analysed using qualitative thematic analysis. These themes were subsequently mapped onto Hoggan’s typology of transformative learning outcomes. CARTA fellows reported shifts in their sense of self; worldviews; beliefs about the definition of knowledge, how it is constructed and evaluated; and changes in behaviour/practices and capacities. This paper argues that the changes described by the CARTA fellows reflect transformative learning that is embedded in CARTA’s Theory of Change. The reported transformation was enabled by a curriculum intentionally designed to facilitate critical reflection, further exploration, and questioning, both formally and informally during the fellows’ PhD journey with the support of CARTA facilitators. Documenting and disseminating these lessons provide a guide for future practice, and educators wishing to revitalise their PhD training may find it useful to review the CARTA PhD curriculum.

## Introduction

The Consortium for Advanced Research Training in Africa (CARTA) was created to address a concern shared by a number of African researchers that research and research training in Africa needed revitalisation. It was premised on the assumption that combining the expertise that exists across the continent with selected non-African partners in a creative cross-institutional and cross-disciplinary way could produce internationally competitive PhD graduates [1].

The CARTA programme is based on the premise that excellent, multidisciplinary research, designed to promote evidence uptake in Africa, led by Africans, will improve public health and population well-being. Based on this assumption, CARTA works to strengthen the capacity of public universities to do excellent research by training and supporting early-career researchers and working with their institutions to create research-conducive environments. In CARTA, we focus on public and population health, but our theory of change applies much more broadly. Importantly, CARTA enrols PhD candidates who are already academic staff of the member institutions so that they can apply their skills in their institution.

**Figure 1.**
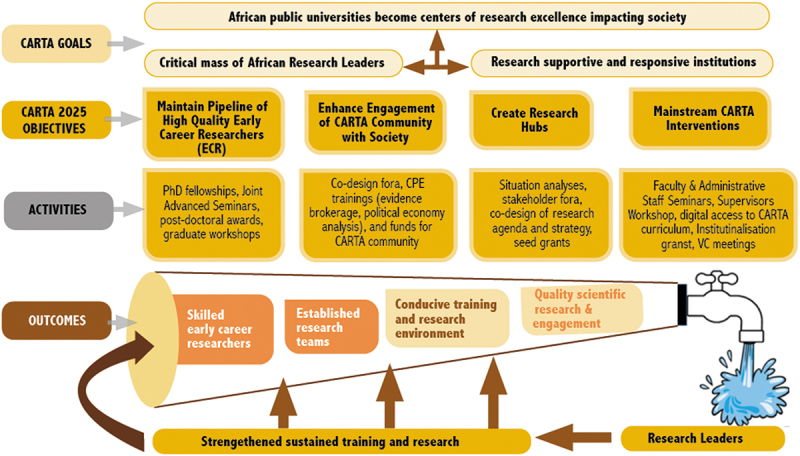
CARTA’s theory of change.

Central to CARTA’s theory of change (see Figure 1) is that motivated excellent researchers acting in unison can be institutional change agents who will train the next generation of African research leaders and engineer institutional shifts towards research-supportive academic environments. However, this will not come about unless there is a conscious effort to train researchers in such a way to instil in them not only skills but also a desire to make an impact at institutional and societal levels. Keeping them motivated is enhanced by having a critical mass of CARTA graduates within institutions who can support each other and an African and international network that CARTA fosters by bringing fellows and facilitators from different institutions together [2].

This paper presents and discusses CARTA fellows’ reported changes they have experienced in their professional lives and worldviews through their participation in the CARTA programme. They describe how they learned to understand, interpret, and do things differently, implying a process of change that was not just about knowledge appropriation.

Transformative learning theory articulates a process where learners experience a change in perspectives that expand and transform their worldviews. Transformative learning is well established and regarded within the adult and continuing education literature; here, we apply it to higher education. The concept of transformative learning posits that the learning experience can promote change by challenging learners to ‘critically question and assess the integrity of their deeply held assumptions about how they relate to the world around them’ [3]. This process is core to the CARTA programme that intentionally challenges PhD fellows from different African universities to critically reflect and assess the teaching and learning approaches used in their universities while at the same time exposing them to alternative approaches to facilitate learning.

Recent studies on the importance of transformative learning in relation to health profession education and research training have highlighted a number of important points around its value in enhancing learning and promoting social change. In their scoping review, Van Schalkwyk *et al*. [4] point to the importance of transformative learning as a theoretical basis for deepening learning and building an understanding of complexity in learning programmes. In particular, the role of transformative learning in promoting new ways of thinking about professional identity is discussed [4–7]. Studies also highlight the importance of building transformative learning approaches into curriculum and pedagogy through opportunities for reflective practice [6,8], creating a context for safe and supportive learning opportunities that encourage critical analysis and varied experience [4,6], and ensuring depth, continuity, and a community of practice [5], authenticity in learner interactions particularly with a community [4,5,9], and opportunities for interprofessional experience [9,10].

While there have been many accounts of transformative learning experiences, missing in the literature are first-hand reports from individuals in Africa who have personally experienced transformative learning either in formal classrooms or in everyday life [11]. This paper analyses CARTA fellows’ perspectives on how the CARTA programme changed their professional lives as researchers and educators. We reflect on how the changes described align with those intended by the learning approaches used in the CARTA curriculum and the notion of creating change agents, which are central to the CARTA theory of change. Documenting and disseminating these lessons provides a guide for future practice [12].

## Methods

### Theoretical approach

In this paper, we draw on theoretical concepts derived from the transformative learning theory proposed by Jack Mezirow [13,14] in which he emphasised the importance of critical thinking, reflection, and challenging assumptions and biases.

Theories of adult learning have included the concept of transformative learning as a process of re-framing and re-interpreting experiences through the development of new perspectives. Mezirow’s theory has been critiqued for its broad focus and attention to individuals rather than social transformation [15]. However, as some argue, social transformation begins with the education of individuals [16]. Mezirow himself responded to the critique by arguing that it is the educator’s role to create the learning conditions that promote critical thinking as a basis for social action rather than promoting social action itself [17]. Thus, ‘*transformative learning refers to processes that result in significant and irreversible changes in the way a person experiences, conceptualizes, and interacts with the world*’ [16]. Other criticisms have focused on Mezirow’s lack of attention to context, including diversity, gender, and sexual orientation [18,19]. While the theory remains controversial, it is nevertheless an important theory that is often cited as a basis for adult education programmes [20].

### Context

The ongoing underfunding of higher education and research in particular has led to the deterioration of African universities, making them unattractive to leading researchers, leading to an exodus of skilled and talented researchers and academics [21]. There is, therefore, a need to strengthen the research infrastructure and culture of African higher education institutions in order to promote research activity and ensure researcher retention. CARTA took this on through a number of institution-building activities that included support to build and equip postgraduate training rooms and libraries and training for academic and non-academic staff in higher education, research management, and supervisory skills. Beyond the institution-building activities, an important part of CARTA was producing a critical mass of PhD graduates who were staff in the consortium universities [2]. CARTA enrolled its first cohort of PhD fellows in 2011, and as of July 2023, 145 fellows had graduated from the programme and another 78 are in the pipeline.

Each cohort of CARTA fellows was composed of fellows from different African universities and disciplines who spent four, face-to-face seminars together at different stages of their PhD journey, each held at different African universities. Each seminar lasted 1 month. The seminars, called Joint Advanced Seminars (JASes), are designed to build fellows’ technical skills, as well as engage them in multidisciplinary experiential and collaborative learning facilitated by CARTA faculty. Through different learning activities and experiences, CARTA PhD fellows are challenged to value evidence and logical argument over position and rhetoric and to value merit. They engage in anti-hierarchical interactions and self-driven learning. They are encouraged to respect themselves and others; embrace a range of approaches to knowledge generation; and challenge prejudice, pre-judgement, and opinions without a basis in evidence. They are challenged to develop a sense of their own agency and a commitment to exercising it and to create a more equal world. The curriculum and pedagogy are described in more detail elsewhere [1] and are available in the public domain through our website cartafrica.org. More information on the PhD training experience as well as CARTA fellows’ PhD experience and the approach to the CARTA PhD curriculum is provided in Supplement 1.

### Data collection

The data analysed and presented in this paper were collected as part of CARTA’s routine monitoring and evaluation. These include routine reports submitted by PhD fellows and graduates. In 2019, a deeper evaluation of the CARTA programme was conducted and a mid-term evaluation report was produced [22]. As part of this exercise, CARTA asked fellows, both those still enrolled as PhD students and those who had graduated, to respond via REDCap [23] to the following question: ‘Given your exposure to CARTA, we ask you to tell us the most significant change that has happened to you (if any) that you think is CARTA’s contribution’. A total of 209 fellows were invited to participate in the CARTA mid-term evaluation.

### Data analysis

Our analysis employed both inductive and deductive approaches [24,25]. True to qualitative research methodology, we initially adopted an inductive thematic approach to the analysis in order to adequately reflect what the CARTA fellows described as changes in their professional lives as researchers and educators. This process involved familiarisation with the data, identifying codes, developing a codebook, and generating themes. This process enabled us to look at a large number of narrative responses to the question posed and organise responses into multiple thematic categories. Responses were coded by three reviewers, and coder agreement was reached by consensus. Since there was only one question, we took the narrative responses directly from the tool and coded them manually. Face-to-face discussions and collaborative work on the coding and analysis took place over the course of a three-day meeting. Further discussions on the data and coding took place at a later meeting where the authors were able to work together for a 2-week period, working with data and focused inductive analysis (Supplement 2). In our attempt to organise and make further sense of the fellow’s descriptions, we adopted a deductive approach to further analyse our data. Transformative learning captured our interest because it resonated with the analysis of our data thus far and with the CARTA theory of change which talks about creating change agents. This paper draws on transformative learning and its adaptations, in particular, the unified or integrated transformative learning theory proposed by Kroth and Cranton [11] and Hoggan’s [16] typology of transformative learning outcomes (see Box 1).

**BOX 1.** Hoggan’s typology of transformative learning outcomes [16].

Worldview	Significant changes in the way the learner understands the world to be. Changes in worldview can be categorised as changes in
	assumptions, beliefs, values, and/or expectations; ways of interpreting experience; more comprehensive or complex worldviews; new awareness and/or understanding.
Self	Any number of ways that learners experience a significant shift in their sense of self:
	self-in-relation to others and/or the world, identity and/or view of self, empowerment and/or responsibility, self-knowledge, personal narrative, meaning and/or purpose, and personality.
Epistemology	A person’s ‘beliefs about the definition of knowledge, how knowledge is constructed, how knowledge is evaluated, where knowledge resides, and how knowing occurs’ [26]:
	more discriminating, utilising extra-rational ways of knowing (e.g. contemplative, spiritual, intuitive, somatic or embodied, emotional, holistic, imaginative, empathetic, artistic, reflective, or multiple ways of knowing), more open, shift in thoughts and/or ways of thinking, more autonomous, more complex thinking.
Ontology	The way a person exists in the world, the overall quality, and tone of one’s existence.
	affective experience of life, ways of being (e.g. more present in the moment or more willing to take chances), attributes (e.g. greater generosity, empathy, or integrity).
Behaviour	Considered an essential component of transformational change, but change but that is associated with at least one other type of outcome
	actions consistent with the new perspective, engaging in social action, changed behaviour, new professional practices, new skills.
Capacity	Learners experience systematic, qualitative changes in their abilities that allow for greater complexity in the way they see, interpret, and function in the world [27].
	cognitive development, consciousness, spirituality.

We mapped the themes identified during the initial analysis of our data to Hoggan’s typology of transformative learning outcomes in order to explore more deeply how the descriptions of change given by the CARTA fellows are captured in the Hoggan typology.

While all four authors of this paper have been engaged with the CARTA programme in different capacities, we deliberately adopted a critical lens in our bid to understand CARTA’s strengths from the perspectives of the beneficiaries. Our motivation for writing this paper was two-fold: first, as educators there was value in understanding the impact of pedagogical experiences on the everyday practices of participants, in particular, a programme that aims to, among other things, graduate change agents. Second, we considered it important to share lessons learned with others interested in capacity-building programmes with similar objectives.

## Findings

Out of the 209 fellows invited to participate in the CARTA mid-term evaluation, 135 of them responded (see Table 1 for the distribution by gender, cohort, and institution).

**Table 1.** Demographics of respondents.

Gender	Responses	Total in CARTA
Female	81	117
Male	54	92
**Cohort**		
Cohort 1	10	20
Cohort 2	7	18
Cohort 3	10	22
Cohort 4	16	27
Cohort 5	14	22
Cohort 6	17	24
Cohort 7	27	26
Cohort 8	19	26
Cohort 9	15	24
Home institution*		
University of Ibadan	24	32
OAU	22	27
Makerere	12	23
University of Rwanda	11	20
Moi University	13	18
University of Nairobi	17	21
University of Malawi**	13	26
Wits	15	26
Dar es Salaam	3	4
APHRC	3	5
Agincourt	0	1
IHI	2	6
**Total**	135	**209**

*University of employment at the time of enrolment in CARTA.

**At the time of the enrolment of the nine cohorts described, the University of Malawi included what is now University of Malawi (UNIMA), Malawi University of Business and Applied Science (MUBAS), and Kamuzu University of Health Sciences (KUHeS).

CARTA fellows reported a number of changes that they attributed to their CARTA PhD experience. The changes are presented under the broad themes outlined in Hoggan’s typology of transformative learning outcomes.

## Changes in worldview

CARTA fellows described how the CARTA experience had changed the way they understood the world to be. These included changes in assumptions, beliefs, values, and/or expectations; ways of interpreting experience; ways of working with others; more comprehensive or complex worldviews; and new awareness and/or understanding.

### Changes in awareness and understanding of issues

*The most significant change that I have experienced is critical thinking. Prior to my contact with CARTA, I used to evaluate things from just one perspective but my contact and training in CARTA gave me insight into more ways of looking at issues*. Male fellow enrolled in 2016 (cohort 6).

### Changes in ways of interpreting experience

*CARTA exposed me to wider thinking in public health, an opportunity to view publications and write them using any public health data, networking opportunities, and funding for research. I have used this leverage to expand my research knowledge and experience and I am currently using it to build capacity and develop early career researchers*. Male fellow enrolled in 2011 (cohort 1)

### Developed more comprehensive worldviews

*Honestly, CARTA training has influenced my ability to critically think on issues and has given me a unique view of perceiving issues. The knowledge and skills have so far given me a platform to make reasonable and meaningful contribution to the best of my understanding within my work environment particularly in teaching and guiding undergraduate students and communication with my colleagues in various activities and also in developing my research work*. Female fellow enrolled in 2017 (cohort 7)

## Changes in self

CARTA fellows described how they had experienced a significant shift in their sense of self including self-in-relation to others and/or the world; identity and/or view of self; empowerment and responsibility; self-knowledge; meaning and/or purpose and personality.

### Changes in view of self

*The CARTA experience was life-changing for me. CARTA inculcated in me the notion that I had been called to be a researcher- a world-class one at that. This meant I had to re-engineer who I was as an individual and commit to a career in research*. Male fellow enrolled in 2014 (cohort 4)

### Shifts in identity and personal narrative

*CARTA has really challenged my way of thinking around research. Prior to becoming part of this fellowship my view of research was narrow, I saw myself as an expert and my participants as subjects whom I could just request to take part in my study to answer the question that I developed based on the literature I reviewed*. Female fellow enrolled in 2019 (cohort 9)

*I have become a better woman, wife, mother, researcher, writer, peer reviewer and academic. I am inspired to do more for my people. Change begins with me!* Female fellow enrolled in 2017 (cohort 7)

### Self-knowledge

*Through CARTA my view of research has changed a great deal. While previously I thought I could do research from a solo approach, I have come to appreciate the role played by a multidisciplinary approach – that I cannot be best in everything*. Male fellow enrolled in 2014 (cohort 4)

### Self-in-relation to others

Not only did fellows report a change in their sense of self but also that this change was affirmed by how they were recognised by others.

*I am also an important resource person whom the department uses to assess the quality of research proposals and dissertations before they are accepted. With this, I have been recently appointed as a coordinator of graduate studies in the department and a program coordinator for the Masters of Research and Public Policy program*. Male fellow enrolled in 2014 (cohort 4)

### Empowerment and responsibility

*Through exposure to CARTA’s pedagogy, my teaching has significantly improved. I have trained a number of postgraduate students who are now either occupying lecturing/research positions or have secured scholarships for doctoral studies in the United States. Due to my many efforts to build the research capacity of graduate students, I was made Chair of the research capacity straightening team in my department*. Male fellow enrolled in 2011 (cohort 1)

## Changes in epistemology

Fellows described changes in their ‘beliefs about the definition of knowledge, how it is constructed, how it is evaluated, where it resides, and how knowing occurs’. They were now more discriminating and were utilising extra-rational ways of knowing.

### More open to new ways of seeing things

*Critical thought was emphasized. From the recruitment phase and through other phases of the fellowship, we were tasked to embrace critical thinking as a scientific value, question things, and be open to learning alternative ways of seeing things*. Male fellow enrolled in 2016 (cohort 6)

### Embraced other ways of knowing

… .*diagnostic sessions, journal clubs, and other group activities within the JASes, have availed me the opportunity to listen to diverse valuable perspectives of both peers and facilitators alike. Hence, I have grown an appreciation for the perspectives and inputs of others*. Female fellow enrolled in 2015 (cohort 5)

### More discriminating and utilising other ways of knowing

*I can now think of different approaches to solving problems as an academic and researcher. In particular, I was able to make a unique and significant contribution to knowledge in my PhD because of the training on critical thinking that I was exposed to through CARTA. Also, I have been able to integrate critical thinking into my teaching method as I involve my students in the teaching-learning process within and outside the classroom*. Male fellow enrolled in 2016 (cohort 6)

*Through CARTA I feel like I finally was able to bring together disparate skill sets (mixed methods) and interests (infant feeding, HIV prevention, capacity strengthening, social determinants of health, and social and behavior change communication) into a research identity that I can build upon professionally. I now have a number of first-authored publications I can point to that clarify my position and interests as a scholar*. Female fellow enrolled in 2015 (cohort 5)

### More complex thinking

*[CARTA] has also exposed me to the interdisciplinarity of research. Many research questions cannot be answered by one approach and this is what I now help my students to take up. To me, the multiple skills approach in the Joint Advanced Seminars were the best in cultivating this in my professional life*. Male fellow enrolled in 2017 (cohort 7)

### More autonomy

Fellows reported being more autonomous and having more self-confidence and agency.

*… CARTA has fostered a global network of fellows and JAS facilitators, many with whom I am collaborating on papers and research. CARTA has built my confidence to initiate ideas and collaborations as a researcher dedicated to the endeavor to increase research generated for and by Africans*. Female fellow enrolled in 2015 (cohort 5)

## Changes in ontology

CARTA fellows described how the CARTA experience had changed their affective experience of life including being more willing to take chances and being researchers of integrity.

### Excellence and collaboration

*The ‘spirit of excellence and selfless giving’ is quite tangible and infectious in CARTA. This is evident in the seminars and sharing of grant opportunities with fellows (current and graduated fellows). This has helped me to also seek ways of improving not just our current PhD students but even those who have graduated to assist them to remain successful in their academic careers*. Female fellow enrolled in 2013 (cohort 3)

### More conscious of integrity and ethical conduct in research

*On being ethical, CARTA made me conscious of ethical issues in the most mundane aspects of research design, with the added enriched knowledge that what we do matters; that we matter, just as much as what we do, and that whether we do it the right or the wrong way may help people or destroy lives*. Male fellow enrolled in 2016 (cohort 6)

### Appreciation of who they were in the world and how they may impact it

Fellows reported a changed appreciation of who they were in the world and how they may impact it.

*Being a CARTA fellow means being articulate, critical, competitive, and ethical. The JAS program, right from JAS 1, is structured to ensure that fellows imbibed these values. We were encouraged to be as precise and concise in how we present ideas and issues, to appreciate the value of precision in getting our thoughts across, and to learn that in many circumstances, we will be communicating with people outside our field of expertise. In short, they made us understand that changing practice and policy requires a strong commitment to clarity of thought*. Male fellow enrolled in 2016 (cohort 6)

### Changes in their way of being

They also reported changes in their way of being that has resulted in changed relationships with others at work.

*CARTA really changed my outlook to life. I feel I can make a unique contribution to humanity through research*. Male fellow enrolled in 2014 (cohort 4)

## Changes in behaviour/practice

While nearly all fellows described changes in their knowledge and skills, this extended to reported changes in behaviour. They reported changes in the way they conducted research, engaged in social actions such as networking, and changes in their professional practice.

### Skills acquired changed improved relationships

*The skills acquired through CARTA have improved my positive relationship and interpersonal interaction with administrative staff in my institution*. Female fellow enrolled in 2015 (cohort 5)

### Changes in research practice

*My exposure to CARTA has been life changing. CARTA reflects in everything I do. Time management, literature search, use of scientific evidence, finding a research gap, achieving milestones in a timely manner, Thinking outside the box ….Everything!*

*I use the skills I have acquired at work, I am more efficient and effective. I use the skills set in my family life as well. As such I have even been able to attain a good work life balance*. Male fellow enrolled in 2017 (cohort 7)

### Changes in professional practice

*Before joining the CARTA program, my teaching was dominantly in form of lectures, especially talk and chalk from the beginning to the end. XX* [removed to maintain anonymity], *which is my home discipline is traditionally characterized by relying heavily on theoretical knowledge and philosophical argument. Through the Joint Advanced Seminars (JASes), I was introduced to new learning facilitation techniques, which I have found effective and I have extensively deployed them in facilitating different courses. […] Students through the end-of-the-semester reflection meeting appreciated that learning has become more flexible, enjoyable, and motivating than before. It is no longer perceived as learning for answering examination questions, but rather developing competencies required to do research and address problems around them*. Male fellow enrolled in 2014 (cohort 4)

### Actions consistent with the new perspective

*The skills I have acquired as part of CARTA are self-revealing wherever I go and whatever I do as part of my day-to-day teaching and learning facilitation. I have come to learn and believe that even without a poster mounted somewhere in the institutions, CARTA will always advertise itself through what the fellows are doing within their institutions*. Male fellow enrolled in 2014 (cohort 4)

### Changes in social action

*CARTA constantly provided opportunities to network and learn from several accomplished professional colleagues and facilitators during the JAS seminars some of whom I am still in touch with. I am now always on the look out for professional colleagues that I can link our PhD students with as I found this very useful for my work and career*. Female fellow enrolled in 2013 (cohort 3)

## Changes in capacity

CARTA fellows described how they have experienced systematic, qualitative changes in their abilities that have allowed for greater complexity in the way they see, interpret, and function in the world.

### Development of critical thinking

*I am six months into the CARTA program and only attended the JAS 1 seminar* [so far]. *I think the greatest influence of CARTA on me is improving my critical thinking and writing skills. This improvement came about from my experiences at the JAS 1 Seminar and the ESE:O* [online writing] *interactions/assignments*. Female fellow enrolled in 2019 (cohort 9)

### Cognitive development

*As a whole, CARTA has helped me develop leadership skills as well as be able to develop my professional development plan and improve my emotional intelligence. … . I did not just earn my PhD but I have also become more motivated and aggressive in participating in research-related activities*. Male fellow enrolled in 2014 (cohort 4)

### Changes in consciousness

*Yes, my experience of and exposure to CARTA has influenced me and my practice in that I am now a better researcher, a better writer, and a better scientist. I am now able to apply the research skills learned to perform better quality research. I am able to supervise my students better in their research projects, approach different types of research with more confidence, write better quality publications and assist my students to do the same*. Female fellow enrolled in 2013 (cohort 3)

## Discussion

CARTA’s Theory of Change is premised on the assumption that fellows in the programme will be catalysts for change in their own institutions, in the wider context of public health research in Africa, and as part of the global community of academic researchers. In order for these elements to occur, a certain amount of individual-level change must occur. Many of the statements by CARTA graduates and fellows can be seen as having depth, breadth, and relative stability [28]. Fellows reported the enduring impact of CARTA on their teaching and research lives in a broad range of contexts. None of this came about by chance. The content, e.g. the journal articles used during journal clubs and how we taught, introduced fellows to points of view that were discrepant with the points of view they held prior to their enrolment. It is this discrepancy between the points of view that they were introduced to and those that they held that led to critical reflection, exploration, questioning, and possibly a shift in perspective.

Fellows reported their greater commitment to quality and integrity as well as striving for greater professionalism in their work. The depth and stability of this transformation are evident as participants feel that they are CARTA’s own best advertisement, reflecting these values in a way that marks them as products of CARTA. We see this in the statements around the recognition of professional skills, the importance of networking and interdisciplinary work, and the way fellows describe their commitment to research in a new light, seeing the value in working with others and sharing their skills. This is particularly evident in the changing commitment to teaching and mentoring the next generation. Fellows shared their new understanding of the value of ensuring that they facilitate change in their own institutions, highlighting the new perspective on their role as teachers and role models.

Fellows described their personal growth fostered by their experiences with CARTA and the skill-building, knowledge, self-awareness, and sense of responsibility to be the best researchers they can be. This transformation was articulated in numerous ways as fellows noted their increased skills in researching, writing, teaching, and mentoring as well as growth in their professionalism and confidence in their own abilities as researchers. Evidence of endurance of such change is reflected in descriptions of roles within university departments, willingness to seek out opportunities, and the recognition that many have received in their own departments and universities. Many described their growing capacity to play a leadership role in their departments and attribute this to the breadth of skills acquired throughout the CARTA programme. These skills also contributed to the individual’s sense of personal and professional development as researchers. Fellows identified specific aspects of CARTA’s approach as key to their development with one of the fellows highlighting the importance of being able to ‘leverage’ skills to continue to improve their work. This aligns with the emphasis in the literature on the value of building transformative opportunities into the curriculum [4,8].

In this area of transformation, we see strong evidence of the continuum as virtually all CARTA fellows who responded, from newly recruited to cohorts of graduates of the programme, and spoke about the development of their writing and critical reading skills as a result of the attention given to this element early in the programme. This transformation begins early and endures after fellows graduate and work in their research fields. As Hoggan and Mezirow have argued, transformation is not a discrete or finite event. Rather, it is an ongoing process, subject to further change, growth, and development of new ideas. There is strong evidence in the data to show that fellows perceive themselves as having developed lifelong skills in the area of academic writing and they continue to hone these skills as they seek to publish their work. From a transformative learning point of view, as Hofer notes [26], we were interested in ways that individuals internalise and understand and embody different kinds of knowledge more broadly. Fellows described the ways in which, as Hofer outlines, they had come to understand a multitude of ways of knowing and were less dependent on a singular scientific approach to questions in research. As noted by Müller J et al. [5], the importance of authentic experiences in the community and with other members of a team enhances the understanding of how knowledge matters. This was particularly evident in comments about newfound respect for and understanding of multi-disciplinarity and teamwork, being part of a network, and gaining the benefit of many perspectives. CARTA fellows and graduates reported changes in worldview in a number of themes that emerged from the study. These include a sense of ‘selfless giving’ as a value, seeing the role that research can play in a changing world, and ‘re-engineering’ themselves to engage with this newfound possibility refers to the way a person exists in the world. This was linked to the importance CARTA placed on ensuring opportunities for developing critical thinking and reflection as part of the learning journey. Sokol et al. [6] highlight the importance of attending to the emotional impact of transformative learning through reflection as it both shakes and shapes perception in practice. Fellows reported a deeper awareness of academic integrity and a realization that they are recognized in their academic realm as having the values espoused by the program in an enduring way.

Such comments speak to the depth and breadth of change when scholars identify how they are associated with CARTA without having to advertise this fact. Much like the skills of building the self in this experience, fellows described how CARTA had also generated opportunities for lasting behaviour changes. Such changes could be linked to the typology through changing teaching methods, a stronger commitment to research, and a commitment to integrity such as recognising plagiarism as inappropriate. Change in behaviour as a learning outcome seemed to be necessary but not sufficient; it was often considered an essential component of transformational change but was always associated with at least one other type of outcome. Fellows described changes in behaviour in the sub-categories of (1) actions consistent with new perspectives, (2) engaging in social action, (3) changed behaviour, (4) new professional practices, and (5) new skills. This aligns with studies describing the importance of threshold concepts in building enduring transformation that alters perceptions [10,29]. Capacity refers to developmental outcomes whereby learners experience systematic, qualitative changes in their abilities that allow for greater complexity in the way they see, interpret, and function in the world [27]. The focus of capacity in Hoggan’s [16] typology is about increasing complexity and consciousness. We see the evidence of this in the commitment to teaching as fellows reported how their understanding of the teaching role had changed towards perpetuating change and promoting enduring practices that will improve research capacity in their institutions. The focus here was the development of greater capabilities and areas of strengthened supervisory, mentorship, research, and leadership skills as a result of the training and experience they had with CARTA. Capacity was also evident when fellows reported about the recognition they received within their own institutions. The capacity to function competently within their professional worlds was often rewarded with responsibility and support within their faculties.

The Hoggan typology allowed us to interrogate in more detail the impact of our curriculum. While we ascribed some quotes to particular aspects of the typology, they were some overlaps reflecting how transformation is multifactorial. This suggests, as is postulated by Mezirow, that a change in self or ontology is required for a change in practice. This analysis confirmed that our approach to teaching is as important as the content, if not more. It also affirmed the CARTA theory of change that investing in change makers is a valid approach to individual and institutional capacity development

While this study was based on a particular population of scholars as part of a larger programme evaluation, it has transferability in that we sought to gather information on the way the scholars themselves understand the impact of the programme and its specific interventions in changing their way of working. The risk of false-positive responses due to fellows still undergoing the programme wanting to please their funders is minimal since participation was voluntary, not linked to any programme support or activity, and treated anonymously. Also, the fellows’ responses linked programme interventions to the changes that they described. They were not asked to go through the programme interventions and bring out their benefits, but were asked to highlight what had changed for them and how the programme had influenced that change. While it is possible, but unlikely, that all non-responders experienced no change attributable to CARTA, this would still not invalidate the findings and conclusions drawn from those who did. This kind of reflexive understanding of the way CARTA has promoted transformative learning has relevance to any programme that seeks to make social change part of its objective.

## Conclusion

This paper documents fellows’ reported changes as a result of engaging with the CARTA Programme. We argue that the reports by the fellows depict changes in fellows’ critical reflection, further exploration, questioning, and shifts in perspective – in sum, transformation. We mapped the concrete examples of growth and change as fellows describe them onto Hoggan’s typology of transformative learning outcomes [16] in a bid to show how CARTA’s theory of change has had a demonstrated impact among fellows in the programme. The CARTA programme was described by fellows as having shaped their personal and professional lives through transformation of perspectives that foster social change. Fellows also note an impact on their institutions’ practices through changes in their roles, the respect they receive from colleagues and supervisors, and the ways in which their own teaching practices had been influenced. In line with CARTA’s objectives of changing the culture of research practice and influencing more widely research excellence within African universities and research institutions, these changes are part of the momentum that CARTA fellows reported as formative and enduring. Educators looking to revitalise their PhD training may consider using and/or adapting the CARTA PhD curriculum.

## Supplementary Material

Supplemental Material

## Paper context

Transformative learning theory remains largely absent in discussions on higher education in Africa. We present and discuss the perspectives of the CARTA fellows on the changes they have experienced in their professional lives and worldviews enabled by their participation in the CARTA programme. Their experiences reflect a transformative learning process enabled by a curriculum intentionally designed to facilitate critical reflection. Such intentionality should be embedded in structured interdisciplinary PhD training across the continent to elicit similar transformative experiences.
