# Supplementary material for: Transformed through the CARTA experience: changes reported by CARTA fellows about their PhD journey
Source: Glob Health Action. 2023 Nov 9;16(1):2272392. doi: 10.1080/16549716.2023.2272392 (PMC10653687; doi:10.1080/16549716.2023.2272392)
Supplement: Supplemental Material [file ZGHA_A_2272392_SM4199.zip › Transformed_through_CARTA_Supplemen_6.docx]

**Supplement 1 CARTA fellows’ PhD experience and the approach to the CARTA PhD curriculum.**

**The CARTA fellows**

The selection of CARTA PhD fellows is rigorous, competitive, transparent, and prioritises scientific excellence, gender parity and equity between member institutions. PhD fellowships are open to Master’s degree holders employed in any one of the CARTA African member institutions (universities and research institutes). Applications are invited from any discipline but the proposed research must be relevant to public and population health. PhD fellows must register at any of the African member universities.

**The CARTA PhD experience**

The selected fellows receive training that is multidisciplinary in nature and complements the usually discipline-specific courses that they receive in their institution of PhD registration. They receive financial support of various kinds including a stipend, research grants, opportunities to attend conferences. They attend four specifically designed Joint Advanced Seminars (JASes) which bring people from different disciplines, institutions and countries together. The Joint Advanced Seminars are residential seminars that take place at key moments of the fellows PhD path and have been optimized, in content and in timing, to maximize the progress of the fellows in their PhD^[[1]](#endnote-1)^. The JASes promote the establishment of mentorship relationships between fellows of the same and different cohorts and the more traditional vertical mentoring between facilitators and fellows^[[2]](#endnote-2)^.

A core component of the CARTA experience is the exposure to learner-centered teaching methods and the interaction with the wider CARTA community that models academic citizenship. Most of the training is based in experiential learning and learning-through-practice. Lectures are used when needed but they are not the gold standard to aspire to. We focus on and model values. Our approach to training uses many interactive and experiential learning methods. We operate from the position that post graduate students learn more from talking to each other than from listening to someone lecturing. The role of a facilitator in this situation is guide what students are talking about. The peer learning environments we create allow for ‘light bulb’ moments as fellows spark off one another. Beyond teaching research methods and analysis, we focus on transferable skills such as academic writing, academic citizenship, critical thinking, leadership, preparing personal development plans. The curriculum is a good complement to discipline specific PhD training.

Our approach to learning is that all learners are more likely to develop productive working relationships with colleagues if they have also interacted in an informal setting, particularly if they had fun. We incorporate this into our curriculum during the second JAS, as one example, beyond more formal mentoring session, supervisors and PhD fellows all attend a dinner together. Seating is assigned in advance to ensure that PhD fellows and their supervisors sit next to each other at the same table and that the table has a spread of institutions and disciplines represented.

A previous publication discusses the curriculum in more detail and is available: *Building the capacity to solve complex health challenges in sub-Saharan Africa: CARTA’S multidisciplinary PhD training* <https://link.springer.com/article/10.17269/CJPH.107.5511>^[[3]](#endnote-3)^ . Additional information about linking teaching with social action is presented in *Universities, the Citizen Scholar and the Future of Higher Education* ^[[4]](#endnote-4)^<https://link.springer.com/book/10.1057/9781137538697>. The CARTA consortium has made its approach to training as well as the PhD and other related curricula available under the Creative Commons CC and can be found on the CARTA website <https://cartafrica.org/teaching-resources/>

1. Ezeh AC, Izugbara CO, Kabiru CW, Fonn S, Kahn K, Manderson L, et al. Building capacity for public and population health research in Africa: The consortium for advanced research training in Africa (CARTA) model. Glob Health Action. 2010;3:1–7. [↑](#endnote-ref-1)
2. Somefun OD, Adebayo KO. The role of mentoring in research ecosystems in Sub-Saharan Africa: Some experiences through the CARTA opportunity. Glob Public Health. 2021 Jan;16(1):36-47. doi: 10.1080/17441692.2020.1776365. Epub 2020 Jun 2. PMID: 32486968. [↑](#endnote-ref-2)
3. Fonn S, Egesah O, Cole D, Giffiths F, Manderson L, Kabiru CW, et al. Building the capacity to solve complex health challenges in Sub-Saharan Africa: CARTA’s multidisciplinary PhD training. Can J Public Heal. 2016;107:e381–e386. [↑](#endnote-ref-3)
4. James Arvanitakis, David J. Hornsby (Eds). Universities, the Citizen Scholar and the Future of Higher Education. Palgrave Macmillan London. 2016. [↑](#endnote-ref-4)
