# Supplementary material for: Transformed through the CARTA experience: changes reported by CARTA fellows about their PhD journey
Source: Glob Health Action. 2023 Nov 9;16(1):2272392. doi: 10.1080/16549716.2023.2272392 (PMC10653687; doi:10.1080/16549716.2023.2272392)
Supplement: Supplemental Material [file ZGHA_A_2272392_SM4199.zip › Transformed_through_CARTA_Supplement_2.docx]

**Supplementary file 2.** Initial inductive analysis.

CARTA fellows reported a number of changes that they attributed to their CARTA experience during the course of their PhD. The changes were categorized into themes that included the development of new networks with other fellows, mentors, and CARTA faculty, which resulted in new collaborations (publishing); development and or enhancement of personal research skills in the areas of literature searching, writing for publication, poster development, grant writing, use of research software, presentations skills; changes in how they now teach and mentor students as a consequence of exposure to CARTA methods and CARTA-facilitated opportunities; growth in confidence as supervisors and in their ability to articulate their own research identity and changes in commitment and attitude. The changes reported by the fellows did not necessarily fit neatly into Hoggan’s typology of transformative learning outcomes. There were however, some similarities, overlaps, and interrelationships between the reported changes and Hoggans’ typologies.

**Developed new networks and knowledge**

Fellows reported that their experience with CARTA was important in fostering both an awareness of the value of networking as well as availing opportunities to develop collaborations and networks as part of a new perspective on knowledge generation. The networking component was linked with success in publication and contributions to problem-solving at the policy and government levels.

*… CARTA has fostered a global network of fellows and JAS facilitators, many with whom I am collaborating on papers and research. CARTA has built my confidence to initiate ideas and collaborations as a researcher dedicated to the endeavor to increase research generated for and by Africans.*

*Through CARTA my view of research has changed a great deal. while previously I thought I could do research from a solo approach, I have come to appreciate the role played by a multidisciplinary approach - that I cannot be the best in everything.*

CARTA fellows described the value of global networking for themselves and their students as they become supervisors. Fellows saw themselves differently in relation to the world of research and academia and reported increases in their abilities and new relationships as part of their way of working.

**Integration of critical thinking and ethics into research practice**

Fellows reported a number of valuable professional skills gained that had enhanced their capacities as researchers, academic writers, and presenters. Most important of these were critical thinking, ethics in research practice, and the capacity for articulating an argument in a concise and precise manner. Perhaps most important was the way such skills as critical thinking and ethical practice have become integrated as part of their practice.

*Being a CARTA fellow means being articulate, critical, competitive, and ethical. The JAS program, right from JAS 1, is structured to ensure that fellows imbibed these values. We were encouraged to be as precise and concise in how we present ideas and issues, to appreciate the value of precision in getting our thoughts across, and to learn that in many circumstances, we will be communicating with people outside our field of expertise. In short, they made us understand that changing practice and policy requires a strong commitment to clarity of thought.*

*The most significant change that I experienced was the ability to think critically. I can now think of different approaches to solving problems as an academic and researcher. In particular, I was able to make a unique and significant contribution to knowledge in my PhD because of the training on critical thinking that I was exposed to through CARTA. Also, I have been able to integrate critical thinking into my teaching method as I involve my students in the teaching-learning process within and outside the classroom*

*Through CARTA I feel like I finally was able to bring together disparate skill sets (mixed methods) and interests (infant feeding, HIV prevention, capacity strengthening, social determinants of health, and social and behavior change communication) into a research identity that I can build upon professionally. I now have a number of first-authored publications I can point to that clarify my position and interests as a scholar.*

Other skills identified included literature searching, writing for publication, poster development, grant writing, the use of research software, and presentation skills. Fellows reported that these skills contributed to the success of their academic career in quantifiable ways such as the number of peer-reviewed publications, number of conference presentations, and number of successful grant applications. Participants identified these skills as being key to identity formation as researchers and scholars going forward.

**Developed new facilitation skills and mentorship approaches**

There were a number of responses that reflected the value and impact of CARTA’s approach to facilitating learning and how this has changed fellows’ approaches to their own teaching, supervision, and mentoring of students. These influences included the transfer and adaptation of classroom facilitation and approaches to strengthened supervision and support for research and methodology.

*Before joining the CARTA program, my teaching was dominantly in form of lectures, especially talk and chalk from the beginning to the end. I now try to make my students part of the knowledge-creation process. We agree together on what is important for them, which forms the basis for developing course content and course outlines. With the use of problem-based learning my classes have tended to be more practical and focus on addressing real-world problems.*

*The CARTA program has enabled me to competently and keenly contribute to the quality of my students’ experience, learning, and outcomes.*

**Confident researchers and supervisors**

Fellows reported changes in their own identities and level of confidence as scholars. They linked this to a sense of personal growth and development fostered through the CARTA program. This building of professional identity was linked to the confidence the fellows felt as a result of their training.

*The CARTA experience was life-changing for me. CARTA inculcated in me the notion that I had been called to be a researcher- a world-class one at that. This meant I had to re-engineer who I was as an individual and commit to a career in research*

*Since 2015 when I became a CARTA fellow, I noticed that my self-confidence has markedly and consistently improved, having been exposed to participatory and interactive learning methods used in the JASes.*

*Yes, my experience of and exposure to CARTA has influenced me and my practice in that I am now a better researcher, a better writer, and a better scientist. I am now able to apply the research skills learned to perform better quality research. I am able to supervise my students better in their research projects, approach different types of research with more confidence, write better quality publications and assist my students to do the same.*

**Recognition of Professional skills in Academic Role (Self and capacity)**

A key change that fellows reported in relation to their role within the university is the recognition from their peers, students, and department heads that they are experts in their field as well as expert teachers and mentors. CARTA graduates are recognized as resources and leaders within their departments and institutions. Participants have been recognized outside the academy through such opportunities as invitations to present at international conferences or to review papers for journals.

*……..CARTA has made me a leader in research at my institution. Due to the skills, I have and the short courses I deliver which are research related, and the research seminars I organize, people recognize* *me as someone who likes research. This has led to more people admiring the CARTA approach."*

*CARTA JASes training has placed me on a platform that has allowed me to impart knowledge and share skills on capacity building learned through the CARTA program…. I have shared the knowledge acquired from JASes training in the research process with both undergraduates and postgraduates. The skills acquired through CARTA have improved my positive relationship and interpersonal interaction with administrative staff in my institution. I have trained doctoral students on how to use EndNote reference manager…, and I shared my skills in the use of e-resources, particularly CIHNARL, Scopus, PubMed for literature search and databases using my institution’s e-library with colleagues.*

*Soon after graduating in 2018, the department trusted my capacity and identified me to teach research courses for PhD and Masters students (which is usually done after 2 years of getting a PhD). I am also an important resource person whom the department uses to assess the quality of research proposals and dissertations before they are accepted. With this, I have been recently appointed as a coordinator of graduate studies in the department and a program coordinator for the Master of Research and Public Policy (MRPP) program.*

*The skills I have acquired as a CARTA fellow are self-revealing wherever I go and whatever I do as part of my day-to-day teaching and learning facilitation. I have come to learn and believe that even without a poster mounted somewhere in the institutions, CARTA will always advertise itself through what the fellows are doing within their institutions.*

For this fellow, the values associated with CARTA have been internalized and changes in perspective have become part of recognizable professional practice.

**Local agents for change mentoring next generation**

A number of fellows identified how they have become stronger academics with specific skills and attributes such as resourcefulness and confidence. Participants also identify ways that they have encouraged their students towards self-improvement through facilitation and engagement in critical approaches in their own work.

*My experience in CARTA has made me a resourceful and confident supervisor to my postgraduate students. I apply the mode of learning I experienced at UofT of students centered learning where I give resources to the students to read and ask them to lead the discussion during class time, instead of using PowerPoint to lecture them.*

*My experience of and exposure to CARTA greatly influenced my teaching and research. Through exposure to CARTA's pedagogy, my teaching has significantly improved. I have trained a number of postgraduate students who are now either occupying lecturing/research positions or have secured scholarships for doctoral studies in the United States. Due to my many efforts to build the research capacity of graduate students, I was made Chair of the research capacity straightening team in my department.*

*I remain an agent of change, an aspiring research leader who is transferring knowledge and skills gained through CARTA to my current and future students."*

**Change in values and attitude**

Fellows reported changes in attitude that resulted in a stronger commitment to research and to professionalism as another significant impact of the CARTA Program. They associated this with the culture of commitment they saw within the CARTA program. Others identified a strengthening of their resolve to be better researchers and teachers to solve problems for their communities and countries.

*On being ethical, CARTA made me conscious of ethical issues in the most mundane aspects of research design, with the added enriched knowledge that what we do matters; that we matter, just as much as what we do, and that whether we do it the right or the wrong way may help people or destroy lives*

*CARTA really changed my outlook to life. I feel I can make a unique contribution to humanity through research.*

*The ""spirit of excellence and selfless giving"" is quite tangible and infectious in CARTA. This was evident in the seminars and sharing of grant opportunities with fellows (current and graduated fellows). This has helped me to also seek for ways of improving not just our current PhD students but even those who have graduated to assist them to remain successful in their academic careers.*

*As a whole, CARTA has helped me develop leadership skills, develop my professional development plan, and improve my emotional intelligence.*

Fellows spoke about leadership as a key value in the CARTA program and something they were now able to embrace as they move forward in their careers.
